# A study into the effect of *Lactobacillus casei* Shirota in preventing antibiotic associated diarrhoea including *Clostridioides difficile* infection in patients with spinal cord injuries: a multicentre randomised, double-blind, placebo-controlled trial

**DOI:** 10.1016/j.eclinm.2021.101098

**Published:** 2021-09-11

**Authors:** Samford Wong, Shashivadan P Hirani, Alastair Forbes, Naveen Kumar, Ramaswamy Hariharan, Jean O'Driscoll, Anand Viswanathan, Graham Harvey, Ravi Sekhar, Ali Jamous

**Affiliations:** a National Spinal Injuries Centre, Stoke Mandeville Hospital, Mandeville Rd, United Kingdom; b School of Health Sciences, City, University of London, London, United Kingdom; c University of Tartu, Estonia, and Norwich Medical School, University of East Anglia, Norwich, United Kingdom; d Midland Centre for Spinal Injury, Robert Jones and Agnes Hunt Orthopaedic Hospital, Gobowen, United Kingdom; e The Princess Royal Spinal Injuries Centre, Northern General Hospital, Herries Rd, United Kingdom; f Department of Microbiology, Stoke Mandeville Hospital, Mandeville Rd, United Kingdom; g Department of Gastroenterology, Stoke Mandeville Hospital, Mandeville Rd, United Kingdom; h Royal Buckinghamshire Hospital, Aylesbury, United Kingdom

**Keywords:** Probiotics, Spinal Cord Injury, Antibiotic Associated Diarrhoea, Malnutrition, Proton Pump Inhibitor, *Clostridioides Difficile*

## Abstract

**Background:**

Antibiotic Associated Diarrhoea (AAD) and *Clostridioides Difficile* Infection (CDI) are of major concern in spinal cord injury (SCI) rehabilitation.

**Methods:**

A multi-centre, randomized, double-blind, placebo-controlled (the ECLISP) trial, was conducted in three tertiary spinal cord injury centre in the UK to assess the efficacy of consuming a probiotic beverage containing at least 6.5 × 10^9^ live *Lactobacillus casei* Shirota (LcS) in preventing AAD and CDI and in patients with SCI and to determine whether proton pump inhibitors (PPI) and under nutrition-risk are risk factors for AAD/CDI. LcS or placebo was given once daily for the duration of an antibiotic course and continued for 7 days thereafter. Follow up was set at 7 and 30 days after the antibiotic course finished. The primary outcome was occurrence of AAD up to 30 days after finishing LcS/placebo. This trial is completed and registered (ISRCTN:13119162).

**Findings:**

Between November 2014, and November 2019, 359 consenting adult SCI patients (median age: 53.3; range: 18–88 years), from 3 SCI centres responsible for providing approximate 45–50% of UK SCI service, with a requirement for antibiotics due to infection were randomly allocated to receive LcS (*n* = 181) or placebo (*n* = 178). Overall, no statistical difference was seen in occurrence of the primary outcomes of AAD at 30 days follow up (45% v 42.1%, RR: 1.071, 0.8-1.4, *p* = 0.639). In the secondary analyses LcS was associated with a lower risk of AAD at 7 (19% v 35.7%, RR: 0.53, 0.29–0.99, *p* = 0.040) and 30 days follow up (28% v 52.2%, RR: 0.54, 0.32–0.91, *p* = 0.015) in the participants who took PPI regularly. Under nutrition-risk was associated with an increased risk of AAD at 7 (RR: 1.76, 1.28–2.44) and 30 days follow up (RR: 1.69, 1.30–2.0). No intervention-related adverse events were reported during the study.

**Interpretation:**

The present study indicates that LcS could not prevent AAD/CDI in unselected SCI patients. LcS might have the potential to prevent AAD in the higher risk group of patients on regular PPI. Confirmatory studies are needed to allow translation of this apparent therapeutic success into improved clinical outcomes.

**Funding:**

Yakult Honsha Co., Ltd.

**Research in context**

**Evidence before this study**

The prevalence of antibiotic associated diarrhoea (AAD) and of *Clostridioides difficile* infection (CDI) in patients with spinal cord injury (PWSCI) patients are reported in range of 14.9 to 30.3%. Probiotics, have been proposed to prevent AAD / CDI by maintain a healthy gut microbiota in hospitalised patients on antibiotic therapy.

**Added value of this study**

Our study identified that the use of a single strain *Lactobacillus casei* Shirota (LcS) supplement could not prevent AAD in PWSCI (44.6% v 42.1%, RR: 1.06, 0.8–1.4; *p* = 0.412) but was associated with a lower risk of AAD at 7 (19% v 35.7%, *p* = 0.040) and 30 days follow up (28% v 52.2%, *p* = 0.015) in participants who took PPI regularly.

**Implication of all the available evidence**

The present study found that LcS could not prevent AAD in PWSCI but has the potential to prevent AAD in the higher risk group of PWSCI on PPI and at risk of under nutrition. Confirmatory studies are needed to allow translation of this apparent therapeutic success into improved clinical outcomes.

Alt-text: Unlabelled box

## 1 Introduction

Antibiotic associated diarrhoea (AAD), described as unexplained diarrhoea that occurs in association with antibiotic administration, is a common complication of antibiotic treatment. It is reported that about 30.3% of patients will develop AAD upon administration of antibiotics [1] but the frequency of AAD can be as high as 60% during hospital outbreaks [2].

Infection with the recently renamed *Clostridioides Difficile* (CDI) occurs most often as a consequence of disruption of the gut microbiota following the use of broad spectrum antibiotics, and is therefore considered the specific cause in a subgroup of AAD. *C. difficile* is a spore-forming, Gram-positive anaerobe. It is a particularly virulent pathogen because it produces an enterotoxin and a cytotoxin, both of which cause mucosal injury and damage to the colon. When antibiotic therapy disrupts this natural defence, *C. difficile* multiplies and produces toxins, causing diarrhoea. In some cases, severe inflammation (*C. difficile* associated colitis) can develop [3].

Probiotics, defined as ‘*live microorganisms which, when administered in adequate amounts, confer a health benefit on the host’*, have been proposed to prevent AAD/CDI by restoring or maintaining a healthy gut microbiome in hospitalized patients on antibiotic therapy, particularly those on broad-spectrum antibiotics [4], but it is still unclear whether specific probiotic strains reduce the overall incidence of AAD/CDI [5], [6], [7], [8], [9], [10].

People with SCI (PWSCI) may be particularly vulnerable to diarrhoea and its consequences because of their long hospital stays for acute care and rehabilitation [11]. In addition, neurogenic bladder dysfunction as a result of SCI often leads to enhanced risk of symptomatic urinary tract infections, due to defective urine storage, bladder stones, foreign bodies or residual urine in the bladder. The use of invasive devices such as urinary catheters further increases the need for antibiotics and the risks of their undesirable effects, including AAD and CDI. Newly injured PWSCI not only have a high risk of upper gastrointestinal haemorrhage, but also usually require anticoagulation therapy to prevent venous thromboembolism. Given these additive risks, most patients are prescribed gastric protection with a proton pump inhibitor (PPI). However, PPI exposure is also a risk factor for AAD / CDI. Literature reports show that patients on PPIs have a relative risk of 1.69 of contracting CDI compared to patients who are not taking the medication [12]. The prevalences of AAD and CDI in PWSCI are reported in the range 14.9 to 30.3% [8,13,14].

Diarrhoea can moreover delay rehabilitation, increase the risk of developing pressure ulcers/delay wound healing and reduce quality of life (QOL). There is growing interest in probiotics to reduce the risk of AAD / CDI. An early study identified the use of PPIs and malnutrition or under nutrition risk [8] as possible risk factors for AAD/CDI, but failed to provide definitive results due to the lack of blinded control data. We therefore planned a randomized double-blind, placebo-controlled study to assess the efficacy of the commercially available probiotic (*Lactobacillus casei* Shirota: LcS) for the prevention of AAD and CDI in adults with SCI. The objectives of the study were: (1) to test the efficacy of daily consumption of a probiotic drink containing at least 6.5 x 10^9^ live LcS in preventing AAD and CDI in SCI patients, and (2) to determine whether (malnutrition or) under nutrition risk and PPI use are a risk factor for AAD/CDI.

## 2 Methods

### 2.1 Study design

This study was a prospective, multi-centre, randomized, double-blind, placebo-controlled study. Patients who had been prescribed antibiotics were identified and approached for consent. After obtaining written informed consent, study data were collected at the time of prescribing antibiotics (baseline) and at follow up, set at 7 days and 30 days after the end of the antibiotic course (Abx+7d, Abx+30d). The study was conducted within the National Health Service in the UK. The three centres involved in this study are responsible for about 45–50% of all specialist SCI service in the UK.

### 2.2 Participants

The inclusion criteria were: patients aged ≥ 18 years, who had sustained a SCI, had been admitted to one of the three investigatory centres, were due to receive antibiotics for an infection, and who were able to take the study drinks within 48 h of the first dose of antibiotic. Patients were excluded from the study for the following reasons: previous recruitment to the study (a patient could not be recruited more than once) and were excluded if they had antibiotic use in the 30 days prior to recruitment – although a single dose of prophylactic antibiotic given 14 to 30 days before recruitment was permitted. Also, patients with diarrhoea within the seven days prior to recruitment, with known gastrointestinal disease that could result in diarrhoea and several other conditions and comorbidities were also excluded. (Supplement table 1)

**Table 1:** Baseline characteristics and outcomes summary.

	Total number with values (missing,%)	LcS group *n* = 181	Placebo group *n* = 178
Age Median (IQ range, range)	351 (8, 2.3%)	53 (28, 19–84)	56 (27, 18–88)
≥65 years (%)		50 (27.6%)	48 (27.0%)
Level of SCI	359 (0, 0%)		
Level of SCI: Tetraplegia (n,%)	178 (0, 0%)	88 (48.6%)	90 (50.6%)
Level of SCI: Paraplegia (n,%)	181 (0, 0%)	93 (51.4%)	88 (49.4%)
Severity of initial neurological deficit	359 (0, 0%)		
AIS^15^ grade A (n,%)	158 (0, 0%)	85 (47.0%)	73 (41%)
AIS grade B (n,%)	57 (0, 0%)	27 (14.9%)	30 (16.9%)
AIS grade C (n,%)	79 (0, 0%)	40 (22.1%)	39 (21.9%)
AIS grade D (n,%)	65 (0, 0%)	29 (16.0)	36 (20.2%)
Centre 1	255 (0, 0%)	129 (71.3%)	126 (70.8%)
Centre 2	69 (0, 0%)	35 (19.3%)	34 (19.1%)
Centre 3	35 (0, 0%)	17 (9.4%)	18 (10.1%)
Mechanical ventilation (n,%)	359 (0, 0%)	15 (8.3%)	22 (12.4%)
Pressure ulcers (n,%)	359 (0, 0%)	63 (34.8%)	56 (31.5%)
History of previous ITU stay (n,%)	359 (0, 0%)	42 (23.2%)	44 (24.7%)
Number of drugs Median (IQ range, range)	348 (11, 3.1%)	9 (6, 1–21)	9 (5, 1–31)
Proton pump inhibitor (PPI) use (n,%)	348 (11, 3.1%)	68 (37.6%)	64 (36.0%)
Multiple antibiotics (n,%)	359 (0, 0%)	55 (30.4%)	71 (39.9%)
Duration of antibiotics Median (IQ range, range)	349 (10, 2.8%)	7 (5, 2–55)	8 (6, 2–69)
High risk antibiotics (n,%)	359 (0, 0%)	94 (51.9%)	106 (59.6%)
Laxative use (n,%)	348 (11, 3.1%)	154 (85.1%)	150 (84.3%)
At undernutrition risk: SNST ≥11 (n,%)	350 (9, 2.5%)	64 (35.4%)	59 (33.1%)
Nil by mouth status (n,%)	355 (4, 1.1%)	2 (1.1%)	5 (2.8%)
Use of enteral feeding tube (n.%)	356 (3, 0.8%)	9 (5.0%)	12 (6.7)
At overnutrition risk: BMI>25 kg/m^2^ (n,%)	343 (16, 4.5%)	78 (43.1%)	81 (45.5%)
Obese: BMI >30 kg/m^2^ (n,%)	350 (9, 2.5%)	32 (17.7%)	34 (19.1%)
Time to take first study drink after first antibiotic dose	355 (4, 1.1%)		
Within 24 h (n,%)	–	137 (75.7%)	145 (81.5%)
24–48 h (n,%)	–	43 (23.8)	30 (16.9%)

SCI: spinal cord injury; SNST: Spinal Nutrition Screening Tool; BMI: body mass index; ITU: intensive therapy unit.

Data are n (%) unless otherwise stated. AIS= American Spinal Injury Association / International Spinal Cord Society neurological stand scale.^15^.

Number used to calculate proportions for other characteristics is proportion of patients with available follow up data.

Abx+7d diarrhoea: occurrence of diarrhoea at 7 days after finished antibiotic course, 7 days after they stop intervention (LcS placebo).

Abx+30d diarrhoea: occurrence of diarrhoea at 30 days after finished antibiotic course, 23 days after they stop intervention (LcS/placebo).

Abx+7d CDI: occurrence of C. diff infection at 7 days after finished antibiotic course, 7 days after they stop intervention (LcS/placebo).

Abx+30d CDI: occurrence of C. diff infection at 30 days after finished antibiotic course, 23 days after they stop intervention (LcS/placebo).

Between November 2014 to November 2018, 359 consenting SCI patients, who were within 48 h of commencing antibiotics, were randomly allocated to receive a fermented milk drink (Yakult®: 65 ml) containing a minimum of 6.5 x 10^9^ colony-forming units (CFU) LcS/bottle, or placebo daily for the duration of the antibiotic course and for 7 days thereafter. The study drink was given at the drug round by nurses. Consumption was monitored on a daily basis by the study team. Minor non-compliance was defined as: two consecutive days of not drinking the study intervention; major non-compliance: three or more consecutive days missed. If participants missed the intervention for more than three days, they were withdrawn from the study.

The participants’ demographics, baseline clinical and nutritional information were collected. These included age, sex, level of SCI and completeness of injury using the International Standards for Neurological Classification of Spinal Cord Injury [15] and cause of SCI. Information about nutritional factors, such as weight and height, route of nutrition, nutrient intake as estimated by food record charts (nil by mouth, less than half, half, more than half, and all eaten), interruptions and supplementation of nutrition (use of oral nutritional supplements and artificial nutrition support), was collected. Additional data, which included the use of mechanical ventilation, the history of intensive care unit stay, the number of medications, the indication, route and the antibiotic used as well as the use of PPIs and laxatives, were recorded.

The perceived risks of the various antibiotics were used to categorise patients into three groups: “low-risk” antibiotics (metronidazole and parenteral aminoglycosides), “medium risk” antibiotics (tetracyclines, sulphonamides and macrolides) and “high risk” antibiotics (aminopenicillins, cephalosporins and quinolones) as described in previous studies [6,8,16] and by the UK National Institute for Health and Care Excellence [17]. (Supplement table 2)

**Table 2:** Summary of severe adverse events.

	Total number with values (missing,%)	LcS group *n* = 181	Placebo group *n* = 178
Severe Adverse Events (%)	359 (0, 0%)	2.2% (4)	3.4% (6)
Unexpected SAE		4	6
Nature of SAE			
Transfer to other hospital (cardiology)			1
High dependency unit admission			3
Intensive Care Unit admission		2	
Death		2	2

### 2.3 Sample size

To estimate the size of the sample, we used data from our previous study [8], where we found a difference of 17.9% between groups in the proportion of patients with diarrhoea (56.3% vs 38.4%), which was used as the target difference for effect size. With alpha=0.05 and power of 90%, we estimated a sample size of 162 per group (in G*Power). After accounting for loss to follow-up at 10% (across the length of the trial), this indicated that 180 participants per trial arm (resulting in a total sample of 360, with equal allocations to groups) would be needed.

### 2.4 Randomization and masking

**Fig. 1:**
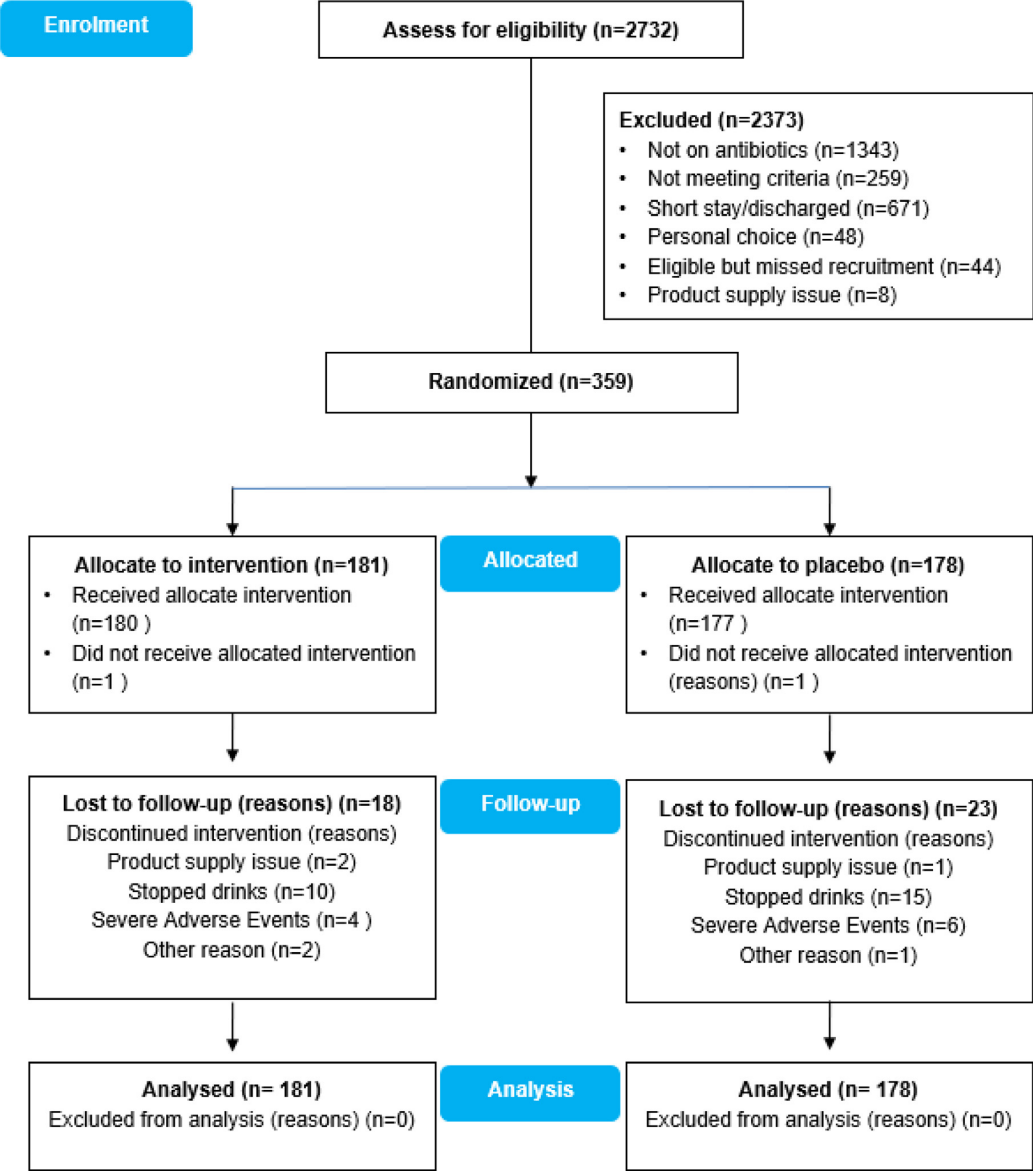
Trial Profile. Decision tree for recruitment and randomization.

A random allocation was carried out by the statistical advisor (SPH) using randomization.com to generate random permutated blocks of 30 for each study site and for previous single dose prophylactic antibiotic use (15 to group 1 and 15 to group 2 per block with no stratification or minimization), until sample size criteria were met. The randomization sequence was concealed from the study team. The study team allocated study drink as per the randomization list and the team was blinded to patient allocation. Emergency unblinding codes were available at individual sites, but these were not used. The randomization list was forwarded to the product supplier (Yakult Europe B.V.) by the study data analyst. The product supplier arranged delivery of product packs to study sites, each with a sticker/code specific for each participant. The placebo was an acidified milk without LcS with nutritional values and organoleptic characteristics (appearance, smell, taste, texture) equivalent to those in the probiotic product. The probiotic drink and the placebo were presented in identical packaging to prevent unblinding by site's staff. Fig. 1, provides the decision tree of recruitment and randomization of subjects.

### 2.5 Primary outcome

The primary outcome was defined as the occurrence of AAD during and up to 30 days after the antibiotic course finished. The bowel movements were monitored routinely by the nursing staff on the ward using the Bristol stool scale [18]. Diarrhoea was defined as more than two liquid stools (Bristol stool scale type 5, 6 or 7) in any 24 hour period.

### 2.6 Secondary outcomes

Whenever diarrhoea was reported, a stool sample was collected and sent to the hospital laboratory for the detection of *C. difficile* toxin. In the present study, CDI was defined by the hospital microbiology laboratory on confirmation of the presence of *C. difficile* toxin, but the method of *C. difficile* toxin detection varied between the laboratories: i.e., screening for glutamate dehydrogenase (GDH) antigen followed by toxin A and B detection, enzyme immunoassays for *C. difficile* toxin A and toxin B, or toxin-producing *C. difficile* gene detection by polymerase chain reaction testing, or a combination of these [19]. The study team recorded the occurrence of diarrhoea throughout the study. The census date was fixed 30 days after the antibiotic course had finished. The secondary outcomes were (1) the occurrence of CDI detected by the study site laboratory and (2) to determine if under nutrition risk and PPI are risk factors for AAD/CDI.

### 2.7 Statistical analysis

The primary statistical analysis was carried out on the basis of intention-to-treat, with all participants being analyzed according to their allocated treatment group irrespective of what treatment they actually received.

Fisher's exact test and χ^2^ test were used to compare rates of diarrhoea, as well as rates of AAD and CDI across categorical variables. Relative risks with 95% confidence intervals, were used to describe the treatment effects of LcS.

A series of screening univariate analyses were undertaken. Logistic regressions were used to establish which factors individually influenced the occurrence of diarrhoea, its duration (and CDI at Abx+7d and Abx+30d follow up). Linear regression was used for continuous outcome measures for duration of diarrhoea and episodes of diarrhoea. Thereafter, statistically significant univariate predictors were used in, multiple binary logistic regression and multiple regression analysis to determine statistically significant predictors for AAD, CDI and other secondary outcomes, after accounting for their relationship to other pertinent variables. Sub-group analyses were performed within treatment arms to analyze the following for association with AAD/CDI occurrence: (i) risk of under nutrition and (ii) use of PPIs. No allowance for multiplicity was made for the secondary outcomes.

To reduce the bias implicit in utilizing only complete cases, multiple imputation procedures for the data was used using the SPSS (SPSS version 25, Inc, Chicago, IL) multiple imputation function with fully conditional specification (maximum iterations of 500) using a Predictive Mean Modeling method to produce 10 imputed datasets. The imputation model included all variables (demographic, clinical and outcomes) involved in the analyses, with PMM imputed variable limits set for imputed values to be within the range of available data. Main outcome variables (i.e. AAD and CDI) were not imputed. These 10 imputed datasets were then individually analyzed as normal; thereafter standard multiple imputation procedures were used to combine the multiple scalar and multivariate estimates quantities [20], [21].

For logistic regression odds ratio (OR), Nagelkerke's R and correctly classified cases are reported. For linear regression, adjusted R^2^ and β coefficients with *t*-test significance are reported. For all tests, a p-value of 0.05 or less, or when the 95% CI for OR did not cross 1.0 were considered as statistically significant. Statistical analysis was performed using the Minitab statistical software (Version 25.0; Minitab, Inc.) and SPSS (Version 19; IBM Corporations).

### 2.8 Ethical consideration

The present study, conducted according to the guidelines laid down in the Declaration of Helsinki, received ethical approval from the National Research Ethics (REC) Committee (reference no. 14/SC/1101) and approval from the local research and development department at each participating site. After the study had been explained by a research coordinator and all questions had been answered, each participant signed an informed consent form prior to trial initiation.

The original study protocol was registered at ISRCTN (IRCTN13119162). The study steering group was set up in July 2014 and the study commenced its recruitment in November 2014. Changes were recommended to the original, approved protocol to improve recruitment specifically in relation to the inclusion and exclusion criteria of patients’ enrolment. The new version of the protocol was developed in accordance with the consolidated standards of reporting trials 2010 guideline and subsequently approved by the sponsor and funder and the REC. [Supplementary File 1]

A register was kept of all patients who withdrew from the study. The reason for consent withdrawal was documented, if provided by the participants, along with the following core patient characteristics: age, gender and level and severity of SCI. To monitor the progress and conduct of the study, all investigators attended meetings before the study and met for communal bi-annual updates and end of study meeting in Jan 2019.

The study was additionally monitored by an external Clinical Research Associate (PHARMExcel) according to applicable provisions of the sponsor's subcontractors monitoring procedures, in conformance with ICH-GCP FDA guidelines, ISO 14,155 and UK-specific laws/regulations.

### 2.9 Definition of under nutrition risk

Participants were considered at risk of under nutrition on the basis of the Spinal Nutrition Screening Tool (SNST) [22]. The SNST assesses eight criteria, of which the majority are recognized as predictors or symptoms of under nutrition: history of recent weight loss; body mass index; level of SCI; presence of co-morbidity; skin condition; appetite; ability to eat. Each step of screening has a score of up to 5 and the total score reflects the participant's degree of risk. A score of 0–10 suggests a low risk, 11–15 a moderate risk and >15 suggests a high risk of under nutrition. Participants who had a SNST score ≤10 were considered at low risk, and all those with a SNST score ≥ 11 were considered at increased risk.

## 3 Role of funding source

This study was funded by Yakult Honsha Co. Ltd. The funder did not have any role in the design of the study, the collection, analysis, or interpretation of the data, the writing of this manuscript, or the decision to submit this manuscript for publication. Dr Samford Wong as chief investigator and corresponding author had full access to the final dataset and made final decision to submit for publication.

## 4 Results

Over the 72 months of the study period, 459 patients were approached by the study team; 48 (10.5%) patients refused to participate in the study, 44 (9.6%) potentially eligible patients were missed because of the tight recruitment timeframe, and eight (1.7%) were excluded as insufficient study drinks were available at the time. Of the 359 patients who met the inclusion criteria and agreed to participate in the study, 41 (11.4%) participants withdrew during the study. The participant flow is summarized in Fig. 1.

The baseline characteristics of the 359 patients (median age: 55, IQ range: 27; 26.7% female; 85.6% Caucasian; 49.6% tetraplegia; 44% complete SCI), and the outcomes summary are summarized in Table 2. SCI was traumatic in origin (*n* = 246, 68.9%) and non-traumatic in 111 case (31.1%). The median onset of SCI was 157 days (IQ range: 2661 days).

The prevalence of risk for under nutrition was 35.1% (*n* = 123) at the time of recruitment and 63 (17.7%) were malnourished on dietitian assessment. We compared participants’ nutrient intake in a small group of selected patients using the food record chart. No difference was observed between the LcS (*n* = 6) and placebo (*n* = 6) groups with regards to the estimated daily intake of energy, protein and dietary fibre. 132 (37.9%) of 348 participants were prescribed PPI (Table 1).

Most participants (64.9%) received one antibiotic as their entry criterion to the study, but 23.4% received two, 7.5% received three and 4.2% received four or more antibiotics. A total of 25 different antibiotics were recorded in the present study: the oral route was used in 46.6% and the intravenous route was used in 53.4% of participants. The median length of antibiotic course was 8 days (IQ range: 5; Range: 2 to 69 days); no statistically significant differences with regard to nature or duration of antibiotic intake were observed between the LcS and the placebo groups. The indications for antibiotic treatment, in descending order, were as follows: urinary tract infections (*n* = 176, 46.1%), respiratory tract infections (*n* = 49, 12.8%), post-operative infections (*n* = 42, 11.0%), sepsis (*n* = 34, 8.9%), pressure ulcers (*n* = 25, 6.5%), wound infections (*n* = 19, 5.0%), cellulitis (*n* = 8, 2.1%), abscess (*n* = 4, 1.0%), toe/finger infection (*n* = 4, 1.0%), osteomyelitis (*n* = 3, 0.8%), tooth infections (*n* = 3, 0.8%) and others (*n* = 15, 3.9%). The number of participants on high-risk antibiotics were closely comparable between the LcS and placebo groups (51.9% in LcS group and 59.6% in placebo group).

At baseline, the LcS and placebo groups were similar with respect to demographic and clinical characteristics, which included: age, onset of SCI, those with tetraplegia and those with complete SCI, the percentage who were on mechanical ventilation, the percentage with pressure ulcers, the percentage with previous history of Intensive Therapy Unit (ITU) stay; laxative use; PPI use, body mass index (BMI), percentage of nil-by-mouth status and use of enteral feeding tubes (Table 1).

Ten (2.8%) severe adverse events were reported in this study. They were not apparently related to the use of investigational product and occurred almost equally in the two groups (LcS: *n* = 4, 2.2% v Placebo: *n* = 6, 3.4%). (Table 2)

## 5 Primary outcome

### 5.1 Antibiotic associated Diarrhoea

The primary analysis was intention to treat and included all patients with available end-point data. The overall prevalence of AAD was 32.1% at 7 days, and 43.6% at 30 days follow up. There was no statistically significant difference between the LcS and placebo groups for the prevalence of AAD at 30 day follow up (45% v 42.1%, RR: 1.071, 95%CI: 0.8 to 1.4, *p* = 0.639). (Table 3)

**Table 3:** Abx+7d diarrhoea: occurrence of diarrhoea at 7 days after finished antibiotic course, 7 days after they stop intervention (LcS placebo) Abx+30d diarrhoea: occurrence of diarrhoea at 30 days after finished antibiotic course, 23 days after they stop intervention (LcS/placebo). Abx+7d CDI: occurrence of C. diff infection at 7 days after finished antibiotic course, 7 days after they stop intervention (LcS/placebo).

*Outcome measures*	Total number with values (missing/359, %)	LcS group	Placebo group	Absolute Risk (95% CI)	Relative Risk (95% CI)	p
**Primary outcome**						
AAD – Abx+30d (n, %)	257 (102, 28.4%)	45% (59 / 131)	42.1% (53 /126)	-0.03 (-0.149 to 0.090)	1.071 (0.810 to 1.415)	0.639
**Secondary outcomes**						
AAD – Abx+7d (n, %)	315 (44, 12.3%)	32.5% (53 /163)	31.6% (48 /152)	-0.009 (-0.111 to 0.093)	1.03 (0.746 to 1.421)	0.858
CDI – Abx+7d (n, %)	275 (84, 23.4%)	7.1% (10 /141)	3.7% (5 /134)	-0.034 (-0.092 to 0.023)	1.901 (0.667 to 5.416)	0.22
CDI – Abx+30d (n, %)	215 (144, 40.1%)	1.8% (2/ 110)	1.9% (2 / 105)	0.001 (-0.047 to 0.050)	0.955 (0.137 to 6.653)	0.963
	Total number with values (missing/359, %)	LcS group Median (IQ range, range)	Placebo group Median (IQ range, range)	Mann Whitney U	z	p
Duration of diarrhoea (days)	258 (101,28.1%)	5 (0, 0-23)	6 (0, 0-31)	8071.5	-0.528	0.597
Episode of diarrhoea	334 (25, 7.0%)	0 (0; 0-7)	0 (0, 0-7)	13420.5	-0.632	0.527

## 6 Secondary outcomes and predetermined subgroup analysis

There was no statistically significant difference between the LcS and placebo groups for the prevalence of AAD at 7 days follow up (32.5% v 31.6%, RR: 1.03, 95% CI: 0.75–1.42, *p* = 0.858) and duration of AAD (median: days, 5 v 6, *p* = 0.597) or the number of episodes of AAD (median, 0 v 0, *p* = 0.527). (Table 3)

### 6.1 Patients on proton pump inhibitors

**Fig. 2:**
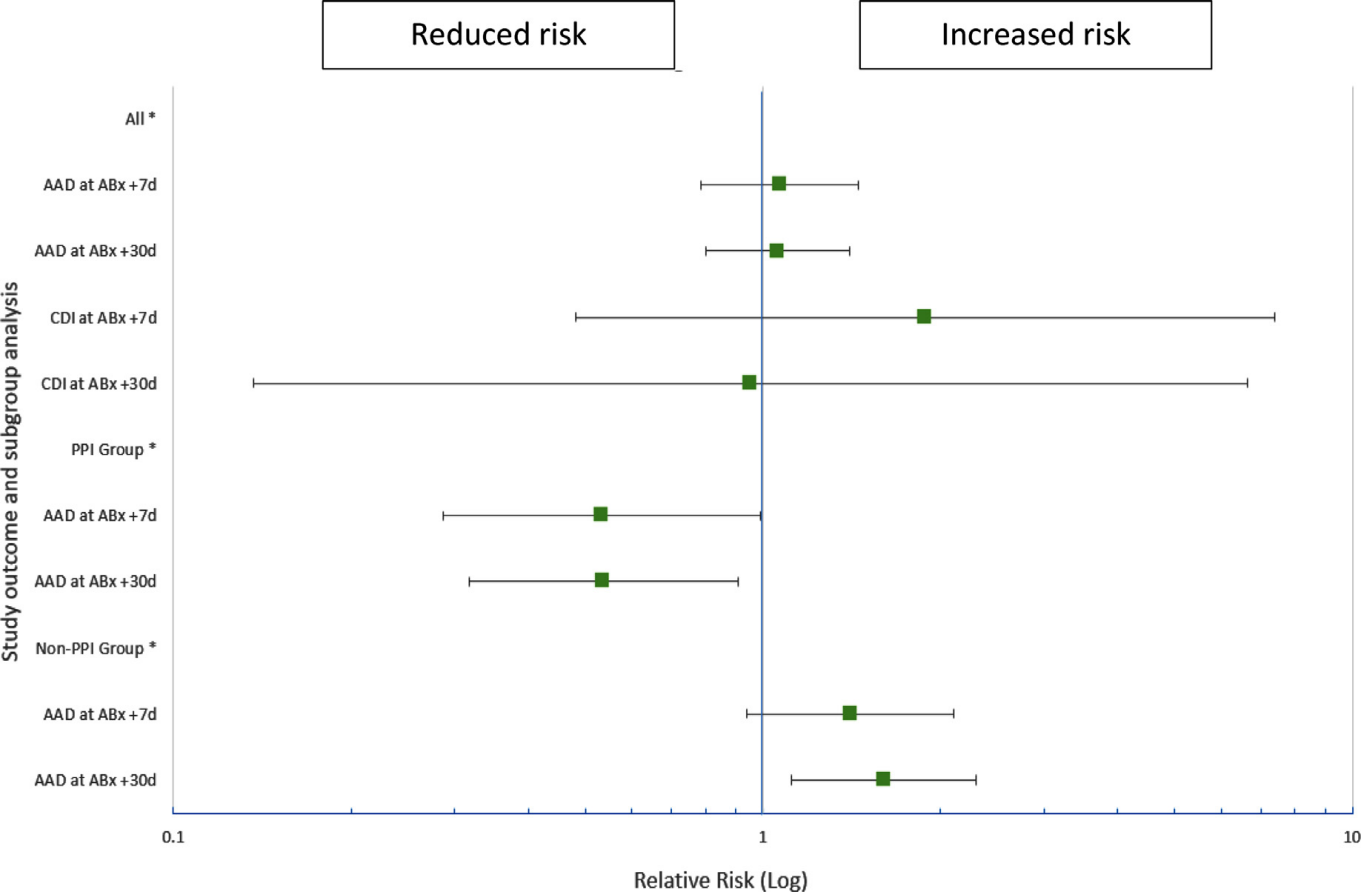
Summary of LcS in study outcome measures, including subgroup analysis.

There was no statistically significant difference between patients on PPI and those who were not, in the prevalence of AAD at Abx+7d (35.7% v 28.6%, χ^2^: 0.823, *p* = 0.36) and AAD at Abx+30d (52.2% v 35.5%, χ^2^: 3.264, *p* = 0.07). However, in the group of patients who were on regular PPI, LcS use was associated with a lower risk of AAD at 7 (19% v 35.7%, RR: 0.53, 95% CI: 0.29–0.99, *p* = 0.040) and 30 days follow up (28% v 52.2%, RR: 0.54, 95% CI: 0.32–0.91, *p* = 0.015) when compared with placebo (Fig. 2 and supplement table 3.1). No difference was observed between LcS and placebo in the incidence of AAD in those patients who did not take PPIs. (Supplement table 3.2)

### 6.2 Malnutrition risk

Overall, participants at malnutrition risk were found to have a significantly higher prevalence of AAD at 7 (43.6% v 24.7%, RR: 1.76, 95% CI: 1.28–2.44, *p* = 0.001) and at 30 days follow up (60.4% v 35.7%, RR: 1.69, 95% CI: 1.30–2.2, *p* < 0.001) Participants who were allocated to LcS or placebo and at risk of malnutrition were found to have a statistically significantly increased prevalence of AAD at Abx+7d and at Abx+30d (Supplement table 3.3 and 3.4)

## 7 Other secondary outcomes

### 7.1 *Clostridioides Difficile* infection

Overall, 15 samples were positive for *C. difficile* toxin at 7 day follow up. The incidence of CDI was 5.5%. There was no statistically significant difference between the LcS and placebo groups at 7 (4.2% v 2.2%, *p* = 0.362) and at 30 days follow up (1.8% v 1.9%, *p* = 0.963) (Table 3)

*Risk factors for antibiotic associated diarrhoea / Clostridioides difficile infection*

The multivariate logistic/regression analysis revealed independent risk factors for AAD at Abx+7d: age older than 65 years, use of high-risk antibiotics, study site and number of drugs.  The risk factors for AAD at Abx+30d were mechanical ventilation, study site and number of laxatives. The risk factors for frequent diarrhoea were age 65 or above, study site, being GDH positive and number of drugs. The risk factors for increased duration of diarrhoea were being nil by mouth, malnutrition as assessed by dietitian, number of drugs and study site. The risk factors for CDI at Abx+7d were being GDH positive and having a history of weight loss, whereas those for CDI at Abx+30d were being GDH positive and having a long duration of antibiotic treatment. Supplement Table 4 provides a summary of the univariate and multivariate results.

## 8 Discussion

This is the first randomised placebo-controlled trial with an adequate sample size in a group of patients with SCI, which has evaluated the efficacy of LcS for the prevention of AAD and CDI. Although this specific probiotic drink could not prevent AAD in all SCI patients, finding suggests that taking LcS is associated with a lower risk of AAD in people with SCI who are on regular PPI therapy.

The present study's conclusion is different from our earlier open-labelled study [8]. The discrepancy in the overall effect may result from the smaller size of the earlier underpowered study (158 vs 359 patients) or from differences in the study design. The present study defined AAD as ≥2 liquid stools (Bristol Stool Scale type 5, 6 or 7) for a 24-hour period, whereas the previous trial required ≥3 days [8]. This altered definition may have led to a failure in distinction between clinically relevant AAD and loose stool due to neurogenic bowel as a result of SCI. Indeed, the definition of AAD varies widely between published studies. For example, Rajkumar et al. [6] defined diarrhoea as ≥2 loose stools, Bristol 6 or 7 a day for ≥3 days, whereas Allen et al. [7] and Helps et al. [9] defined diarrhoea from ≥3 loose stools, Bristol 5, 6 or 7 in a single 24-hour period. The definition for CDI also varies. Using more standardised definitions of study outcomes in AAD/CDI is now improving the quality of and aiding interpretation of newer research studies, especially important for systematic reviews and meta-analyses.

`The inclusion of patients treated in different SCI centres could be considered a strength, but can also be regarded as a weakness. Infection control policies, AAD/CDI definition and antibiotic management were different in the participating SCI centres, thus the influence of these factors on the study results could not be excluded.

The present study suggests malnutrition risk could increase the incidence of AAD in 30 days follow up (60.4% v 37.5%, *p* < 0.01). These results are in agreement with results of a similar published study [8]. Previous research has suggested that consumption of oral nutritional supplements in patients with known malnutrition risk could improve clinical outcomes [23]. Clinicians, therefore should be aware of the need to identify and treat malnutrition which will prevent malnutrition related complications [24], [25].

The present study's incidence of AAD (32.1%) is similar to some previous studies in patients with SCI (14.9–36%) [6,7,8] and seems higher than studies conducted in general populations (10.8 – 17.9%) [6,7]. This may be attributed to a longer follow-up period (30 days) than in many of the other published trials (often only 7–14 days) [6]; diarrhoea may occur up to 2 months after discontinuing antibiotic treatment [26].

Current evidence remains unclear in whether probiotics could reduce the incidence of AAD/CDI in general hospitalised populations [5,6,7]. The complexity of probiotic use is not just strain-, product-, dose- and disease specific, but also includes defining when the probiotic should be administered and the duration of its use; all need to be considered. The present study dose of a minimum of 6.5 x 10^9^ CFU LcS was selected based on the previous trial's data [8]. LcS is well tolerated in clinical settings and has been used in a broad range of patients [27,28]. However, dose and type of probiotic vary between published studies. For example, Allen et al. [7] used a mixed strains probiotic (L. *acidophilus* CUL60, CUL21, *B. bifidum* CUL20, *B. lactis* CUL34 in 6 x 10^9^ CFU /day), Helps et al. [9] used a single strain probiotic (LcS in 13 x 10^9^ CFU /day), and Rajkumar et al. [6] used mixed strains (L. *casei* immunitas DN-114,001 in 10 x 10^9^ CFU /day) as did Selinger *et al* [10] (VSL#3 in 900 x 10^9^ CFU /day).

The compliance with LcS therapy in the present study was good (92.4%), with no known severe adverse events directly related to LcS. It is possible that a higher dose might have yielded a greater benefit. However, the effect of increasing dosage of probiotics should be monitored carefully as unexpected adverse events may also occur.

Another important criterion for any probiotic is that the strains used survive the passage through the stomach and arrive in a viable state in the small intestine and colon. LcS has been shown to survive and be well tolerated in the upper gastrointestinal tract and reach the large intestine in a viable state [29], [30].

The present study does not provide support for a general use of LcS to prevent AAD in SCI patients but suggests that LcS may help to prevent AAD in SCI patients taking PPI regularly. Confirmatory studies are now needed to allow translation of this apparent therapeutic success into improved clinical outcomes. Further research should define the most appropriate target populations. Study designs using standardized definitions of AAD and CDI will help to evaluate the effect of probiotics in preventing AAD/CDI as well as in providing more homogeneous evidence for future systematic review and meta-analysis.

## Contributors

SW- study chief investigator, protocol development, data collection, data analysis, manuscript preparation

SPH- protocol development, data evaluation, statistical support / analyst, manuscript revision

AF – protocol development, data evaluation, gastroenterology adviser, manuscript revision

NK – site principal investigator, protocol development, manuscript revision

RH – site principal investigator, data evaluation, gastroenterology adviser, manuscript revision

JOD – protocol development, clinical supervision, validation and guardian of laboratory data, manuscript revision

AV- data collection, data evaluation, manuscript revision

GH- protocol development, validation and guardian of laboratory data, manuscript revision

RS – protocol development, data evaluation, manuscript revision

AJ – protocol development, clinical supervision, data evaluation, manuscript revision

## Declaration of Competing Interest

Dr Samford Wong (Chief Investigator) has received funding support from Yakult Honsha Co Ltd. Yakult Europe B.V. provided free LcS/Placebo and the necessary equipment to maintain the cold chain. Prof Alastair Forbes receives honoraria form Fresenius Kabi, Takeda and Dr Falk Pharma. All the other authors have no conflicts to declare.

## Appendix Supplementary materials

Image, application 1
