# Supplementary material for: A study into the effect of Lactobacillus casei Shirota in preventing antibiotic associated diarrhoea including Clostridioides difficile infection in patients with spinal cord injuries: a multicentre randomised, double-blind, placebo-controlled trial
Source: eClinicalMedicine. 2021 Sep 11;40:101098. doi: 10.1016/j.eclinm.2021.101098 (PMC8435694; doi:10.1016/j.eclinm.2021.101098)
Supplement: Supplementary file 1 [file mmc1.docx]

**Supplement table 1. Eligibility of subjects for participation in the ECLISP study.**

**Inclusion criteria Exclusion criteria**

1. Subject >18 years of age 1. Re-recruit patient

2. Sustained a spinal cord injury 2. Antibiotic for prophylaxis up to 14 days prior to recruitment

3. Likely to remain in-patient > 6 weeks 3. Diarrhoea within the preceding 7 days prior to recruitment

4. Due to receive antibiotic for infection 4. Bowel pathology that could result in diarrhoea

5. Bowel surgery in last 6 months

6. Infective endocarditis

7. Active inflammatory bowel disease

8. Pancreatitis

9. Regular probiotic use in the previous 8 weeks

10. Antibiotic use in the 30 days before intervention

11. Immunosuppression

12. Nil by mouth / non-functioning gut

13. Known cow’s milk protein intolerance

14. Psychiatric / cognitive conditions that may interfere the study

15. Those unable to give informed consent due to cognitive impairment

16. Pregnant or breastfeeding women

17. Prisoners

Supplement table 2 – Antibiotics risk table for *Clostridioides difficile* associated diarrhoea / antibiotic associated diarrhoea

| **No** | **Name** | **Risk of CDI** | **n** |
| --- | --- | --- | --- |
| **1** | Gentamicin | Low | 92 |
| **2** | Trimethoprim | Low | 70 |
| **3** | Tazocin | Medium | 67 |
| **4** | Nitrofurantoin | Low | 62 |
| **5** | Flucloxacillin | Low | 43 |
| **6** | Teicoplanin | Low | 32 |
| **7** | Ciprofloxcin | High | 22 |
| **8** | Amoxicillin | High | 23 |
| **9** | Metronidazole | Low | 22 |
| **10** | Meropenem | Medium | 15 |
| **11** | Phenoxymethypenicillin | Low | 15 |
| **12** | Clarithromycin | Low | 11 |
| **13** | Temocillin | Low | 10 |
| **14** | Pivecillinam | Low | 9 |
| **15** | Doxycycline | Low | 9 |
| **16** | Co-Amoxiclav | High | 7 |
| **17** | Clindamycin | High | 7 |
| **18** | Vancomycin | Low | 7 |
| **19** | Erythromycin | Low | 3 |
| **20** | Fucidic acid | Low | 2 |
| **21** | Cephalexin | High | 2 |
| **22** | Linezolid | Low | 1 |
| **23** | Ceftriaxone | High | 1 |
| **24** | Amikacin | Low | 1 |
| **25** | Ceftazidime | High | 1 |

Supplement table 3.1 Risk of antibiotic associated diarrhoea in patients taking proton pump inhibitor regularly

|  | **LcS, PPI (Yes)*** | **Placebo, PPI (Yes)*** | **Subtotal** |
| --- | --- | --- | --- |
| **Abx+7d (Yes)** | 12 | 20 | 32 |
| **Abx+7d (No)** | 51 | 36 | 87 |
| 19% vs 35.7%, χ^2^: 4.19, p=0.041 | | | |
|  | LcS, PPI (Yes) | Placebo, PPI (Yes) | Subtotal |
| **Abx+30d (Yes)** | 14 | 24 | 38 |
| **Abx+30d (No)** | 36 | 22 | 58 |
| 28% vs 52.2%, χ^2^: 5.85, p=0.016 | | | |

Supplement table 3.2 Relative risk and number need to treat for AAD/CDI.

|  |  | **LcS group** | **Placebo group** | **Absolute Risk (95% CI)** | | Relative Risk (95% CI) | | **z** | p value |
| --- | --- | --- | --- | --- | --- | --- | --- | --- | --- |
| **All** | AAD at Abx+7d | 32.5% (53 /163) | 31.6% (48 /152) | -0.009 | (-0.1111 to 0.093) | 1.030 | (0.746 to 1.421) | 028 | 0.858 |
|  | AAD at Abx+30d | 45% (59 / 131) | 42.1% (53 /126) | -0.030 | (-0.149 to 0.090) | 1.061 | (0.801 to 1.404) | 0.4 | 0.639 |
|  | CDI at ABx +7d | 7.1% (10 /141) | 3.7% (3 /134) | -0.034 | (-0.092 to 0.023) | 1.901 | (0.667 to 1.421) | 0.9 | 0.346 |
|  | CDI at ABx +30d | 1.8% (2/ 110) | 1.9% (2 / 105) | 0.001 | (-0.047 to 0.050) | 0.955 | (0.137 to 65.416) | 1.2 | 0.965 |
| **PPI (YES)** | AAD at Abx+7d | 19.0% (12 / 63) | 35.7% (20 /56) | 0.167 | (0.007 to 0.319) | 0.533 | (0.287 to 0.990) | 2.1 | 0.0402 |
|  | AAD at Abx+30d | 28.0% (14/50) | 52.2% (24/46) | 0.242 | (0.046 to 0.415) | 0.537 | (0.318 to 0.906) | 2.4 | 0.015 |
| **PPI (No)** | AAD at Abx+7d | 40.2% (39/97) | 28.6% (26/91) | -0.116 | (-0.248 to 0.020) | 1.407 | (0.938 to 2.111) | 1.7 | 0.095 |
|  | AAD at Abx+30d | 60.0% (45/79) | 35.5% (27/76) | -0.214 | (-0.357 to -0.057) | 1.603 | (1.120 to 2.295) | 3.1 | 0.002 |

Supplement table 3.3 Undernutrition risk and risk of antibiotic associated diarrhoea in placebo group

|  | **Placebo, SNST≥11**  **At risk** | **Placebo, SNST ≤10**  **Low risk** | **Subtotal** |
| --- | --- | --- | --- |
| **Abx+7d diarrhoea (Yes)** | 21 | 24 | 45 |
| **Abx+7d diarrhoea (No)** | 32 | 71 | 103 |
| 39.6% vs 25.3%, χ^2^: 3.32, p=0.069 | | | |
|  | Placebo, SNST≥11  At-risk | Placebo, SNST**≤10**  Low risk | Subtotal |
| **Abx+30d diarrhoea (Yes)** | 32 | 30 | 62 |
| **Abx+30d diarrhoea (No)** | 21 | 50 | 71 |
| 60.4% vs 37.5%, χ^2^: 6.7, p=0.0096 | | | |

AAD: antibiotic associated diarrhoea.

Abx+7d diarrhoea: occurrence of diarrhoea at 7 days after finished antibiotic course, 7 days after they stop intervention (LcS/placebo)

Abx+30d diarrhoea: occurrence of diarrhoea at 30 days after finished antibiotic course, 23 days after they stop intervention (LcS/placebo).

LcS: *Lactobacillus casei* Shirota; PPI: proton pump inhibitor; SNST: Spinal Nutrition Screening Tool

Supplement table 3.4 Undernutrition risk and risk of antibiotic associated diarrhoea in LcS group

|  | **LcS, SNST≥11**  **At risk** | **LcS, SNST ≤10**  **Low risk** | **Subtotal** |
| --- | --- | --- | --- |
| **Abx+7d diarrhoea (Yes)** | 27 | 25 | 52 |
| **Abx+7d diarrhoea (No)** | 30 | 78 | 108 |
| 47.3% vs 24.23%, χ^2^: 8.93, p=0.0028 | | | |
|  | LcS, SNST≥11  At risk | LcS, SNST **≤10**  **Low risk** | Subtotal |
| **Abx+30d diarrhoea (Yes)** | 32 | 26 | 58 |
| **Abx+30d diarrhoea (No)** | 21 | 51 | 72 |
| 60.4% vs 33.8%, χ^2^: 8.99, p=0.003 | | | |

AAD: antibiotic associated diarrhoea.

Abx+7d diarrhoea: occurrence of diarrhoea at 7 days after finished antibiotic course, 7 days after they stop intervention (LcS/ placebo)

Abx+30d diarrhoea: occurrence of diarrhoea at 30 days after finished antibiotic course, 23 days after they stop intervention (LcS/placebo).

LcS: *Lactobacillus casei* Shirota; PPI: proton pump inhibitor; SNST: Spinal Nutrition Screening Tool

Supplement table 4: Independent predictors of AAD, CDI, duration and frequency of diarrhoea.

|  | Outcome | Abx+7d diarrhoea | Abx+30d diarrhoea | Abx+7d CDI | Abx+30d CDI | ^**^Episodes of diarrhoea | ^**^Days of diarrhoea |
| --- | --- | --- | --- | --- | --- | --- | --- |
|  | No. of sig. predictors / Total No. of predictors | 5/18 | 3/14 | 2/4 | 2/3 | 4/19 | 5/13 |
| Model Statistics | Nagelkerke’s R - | 0.3581 | 0.3592 | 0.3505 | 0.5964 | - | - |
|  | Correctly Classified cases - STEP 0 - | 65.71% | 56.64% | 96.76% | 98.14% | - | - |
|  | Correctly Classified cases - STEP 1 – | 76.67% | 73.67% | 96.73% | 99.06% | - | - |
|  | Adjusted R^2^ | - | - | - | - | 0.2538 | 0.276 |
| 1 | Age 65 or above | ⯅ 2.917  (1.528- 5.567) | ○ | - | - | ⯅0.511  (0.118-0.904) | ○ |
| 2 | Antibiotics risk highest | ⯅1.937  (1.010-3.717) | ○ | - | - | ○ | ○ |
| 3 | Study centre=1 | ⯅ 15.499  (4.152-7.855) | ⯅13.715  (3.467-54.260) | - | - | ⯅ 1.242  (0.666-1.819) | ⯅1.978  (0.474-3.482) |
|  | Study centre=3 | ⯅16.260 (3.223-82.02) | ⯅24.508  (4.786-125.490) | - | - | ⯅1.036  (0.250-1.822) | ⯅3.303  (1.395-5.212) |
| 4 | High Tetraplegic (C1-C4, Y:1, N:0)(1) | ○ | - | - | - | - | - |
| 5 | Ventilated (Y: 1, N:0) | ○ | ⯅3.353  (1.148-9.798) | - | - | ○ | ○ |
| 6 | Presence of pressure ulcers (Y:1, N:0) | ○ | ○ | - | - | ○ | ○ |
| 7 | Drinks commence – within 24 hours | - | ○ | - | - | ○ | - |
|  | Drinks commence – between 24-48 hours | - | ○ | - | - | ○ | - |
| 8 | Route of antibiotics: ORAL (Y:1, N:0)(1) | - | ○ | - | - | - | - |
| 9 | Route of antibiotics: IV (Y:1, N:0) | ○ | ○ | - | - | ○ | - |
| 10 | Line and Tube (Y: 1, N:0) | ○ | - | - | - | ○ | - |
| 11 | Use of anti-diarrhoea agent (Y:1; N:0) | ○ | - | - | - | ○ | - |
| 12 | Refer to Dietitian (Y: 1; N:0) | ○ | ○ | - | - | ○ | ○ |
| 13 | Comorbidity (Y:1, N: 0) | ○ | - | - | - | ○ | - |
| 14 | Nil-by-mouth (Y:1; N:0) | ○ | - | - | - | ○ | ⯅10.350  (5.416-15.284) |
| 15 | Malnutrition Assessment (Y:1, N:0) | ○ | ○ | - | - | ○ | ⯅2.033  (0.451-3.614) |
| 16 | SNST Category (<11:0; =>11=1) | ○ | ○ | - | - | ○ | ○ |
| 17 | GDH-positive | - | - | ⯅12.793  (2.156-75.92) | ⯅282.567  (3.124-25559.65) | ⯅1.042  (0.324-1.761) | - |
| 18 | No. of drug | ⯅1.169  (1.075-1.270) | - | - | - | ⯅0.066  (0.020-0.111) | ⯅ 0.235  (0.096-0.374) |
| 19 | No. of antibiotics | ○ | ○ |  | ○ | ○ | ○ |
| 20 | Length of Antibiotics incl. in trial (d) | ○ | - | ○ | ⯅1.119  (1.003-1.248) | ○ | - |
| 21 | Number of laxatives | - | ⯅1.568  (1.154-2.132) | - | - | - | - |
| 20 | Fluid Intake (ml/d) | - | - | ○ | - | - | ○ |
| 23 | Additional fluid (ml) | ○ | ○ | - | - | ○ | - |
| 24 | Weight loss % | - | - | ⯅1.145  (1.010-1.299) | - | - | - |

^*^Logistic regression, ^**^Multiple regression; ^⯅^increased chance of event, IV: intravenous

**^○^** Significant in univariate analysis, but not significant after entered in multivariate analysis; **^-^** Not significant in univariate and therefore did not entered in the multivariate analysis

Abx+7d diarrhoea: occurrence of diarrhoea at 7 days after finished antibiotic course, 7 days after they stop intervention (LcS/placebo)

Abx+30d diarrhoea: occurrence of diarrhoea at 30 days after finished antibiotic course, 23 days after they stop intervention (LcS/placebo).

Abx+7d CDI: occurrence of C. diff infection at 7 days after finished antibiotic course, 7 days after they stop intervention (LcS/placebo)

Abx+30d CDI: occurrence of C. diff infection at 30 days after finished antibiotic course, 23 days after they stop intervention (LcS/placebo).
